# Supplementary material for: Causes and consequences of sex-chromosome turnovers in Diptera
Source: Evol Lett. 2026 Mar 12;10(3):253–68. doi: 10.1093/evlett/qrag003 (PMC13231274; doi:10.1093/evlett/qrag003)
Supplement: qrag003_Supplemental_Files [file qrag003_supplemental_files.zip › Supplementary_Datasets.zip!/Supplementary_Datasets/Dataset_1_Heatmaps_ME_enrichment/module1Muscidae_Hydrotaea_cyrtoneurina.pdf]

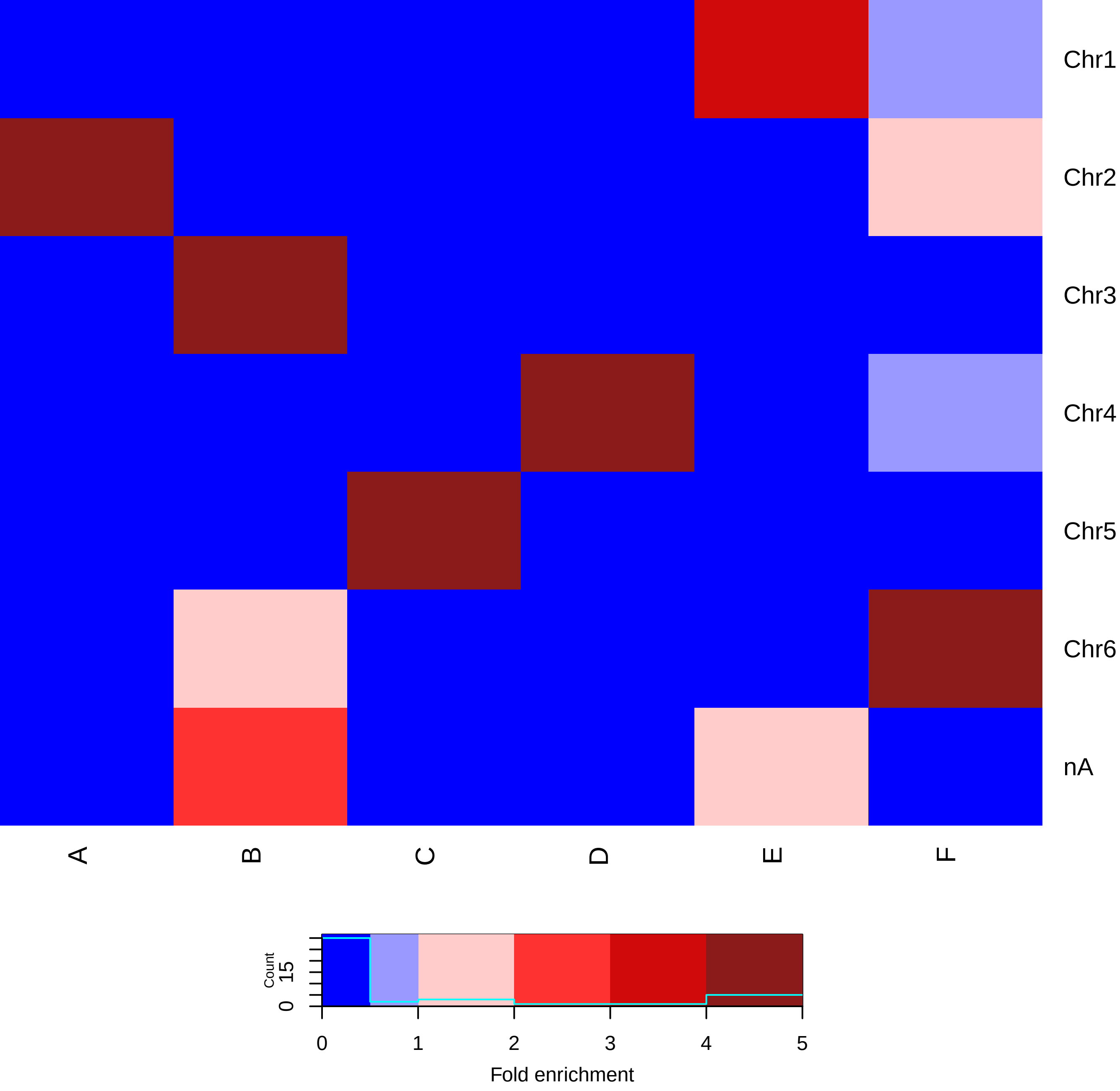

Chr1
Chr2
Chr3
Chr4
Chr5
Chr6
nA
A
B
C
D
E
F
Count
0
15
0
1
2
3
4
5
Fold enrichment
